# Arnold-Chiari Malformations in Pregnancy and Labor: Challenges and Management Strategies

**DOI:** 10.7759/cureus.43688

**Published:** 2023-08-18

**Authors:** Aditi Mishra, Shoyeb Hirani, Sajid Hirani, Mohammed Yusuf D Shaikh, Shubham Khanholkar, Roshan Prasad, Mayur Wanjari

**Affiliations:** 1 Medicine, Jawaharlal Nehru Medical College, Datta Meghe Institute of Higher Education and Research, Wardha, IND; 2 Medicine, Mahatma Gandhi Medical College and Hospital, Aurangabad, IND; 3 Internal Medicine, Jawaharlal Nehru Medical College, Datta Meghe Institute of Higher Education and Research, Wardha, IND; 4 Research and Development, Jawaharlal Nehru Medical College, Datta Meghe Institute of Higher Education and Research, Wardha, IND

**Keywords:** anesthetic considerations, symptom relief, multidisciplinary approach, management strategies, challenges, labor, pregnancy, arnold-chiari malformations

## Abstract

Arnold-Chiari malformations (ACMs) present unique challenges in pregnancy and labor, requiring a comprehensive understanding and multidisciplinary approach to care. This review article provides an overview of ACMs, including their definition, classification, and prevalence. The challenges in diagnosing ACMs during pregnancy, the available imaging modalities, and screening recommendations are discussed. The impact of ACMs on maternal health, fetal development, and the management strategies employed during pregnancy and labor are explored. Emphasis is placed on the importance of a multidisciplinary approach involving neurologists, obstetricians, and other specialists. Medical management options for symptom relief, surgical interventions, and anesthetic considerations during labor and delivery are also addressed. The importance of postpartum care, breastfeeding considerations, and long-term follow-up for women with ACMs who desire future pregnancies are highlighted. Finally, areas for further research and advancements in ACM management are identified. By improving our understanding and management of ACMs in pregnancy and labor, healthcare professionals can optimize care and improve outcomes for mothers and babies affected by this condition.

## Introduction and background

Arnold-Chiari malformations (ACMs) are a group of neurological conditions characterized by structural defects in the base of the skull and the cerebellum. These malformations can cause various symptoms, including headaches, difficulty swallowing, dizziness, and coordination problems. ACMs are typically present from birth but may not be diagnosed until later in life [1,2]. ACMs present unique challenges and considerations for both the mother and the developing fetus in pregnancy and labor. Pregnancy itself can exacerbate symptoms associated with ACMs due to hormonal changes, increased intracranial pressure, and alterations in blood volume. Additionally, the physiological stress of labor and delivery can further complicate the management of ACMs [3-5].

This review article aims to provide a comprehensive overview of the challenges and management strategies related to ACMs in pregnancy and labor. By examining the existing literature and current practices, we aim to enhance the understanding of healthcare professionals caring for pregnant women with ACMs. The article will explore the impact of ACMs on maternal health, fetal well-being, and the management options available to optimize outcomes for both mother and baby. Additionally, we will discuss the importance of a multidisciplinary approach in managing ACMs during pregnancy and labor. Through this review, we hope to contribute to the existing body of knowledge on ACMs in the context of pregnancy and labor, ultimately improving the care provided to women with this condition. By addressing the unique challenges and outlining effective management strategies, healthcare professionals can enhance their ability to navigate the complexities of managing ACMs during pregnancy and labor, leading to improved outcomes for both mother and child.

## Review

Methodology

**Figure 1:**
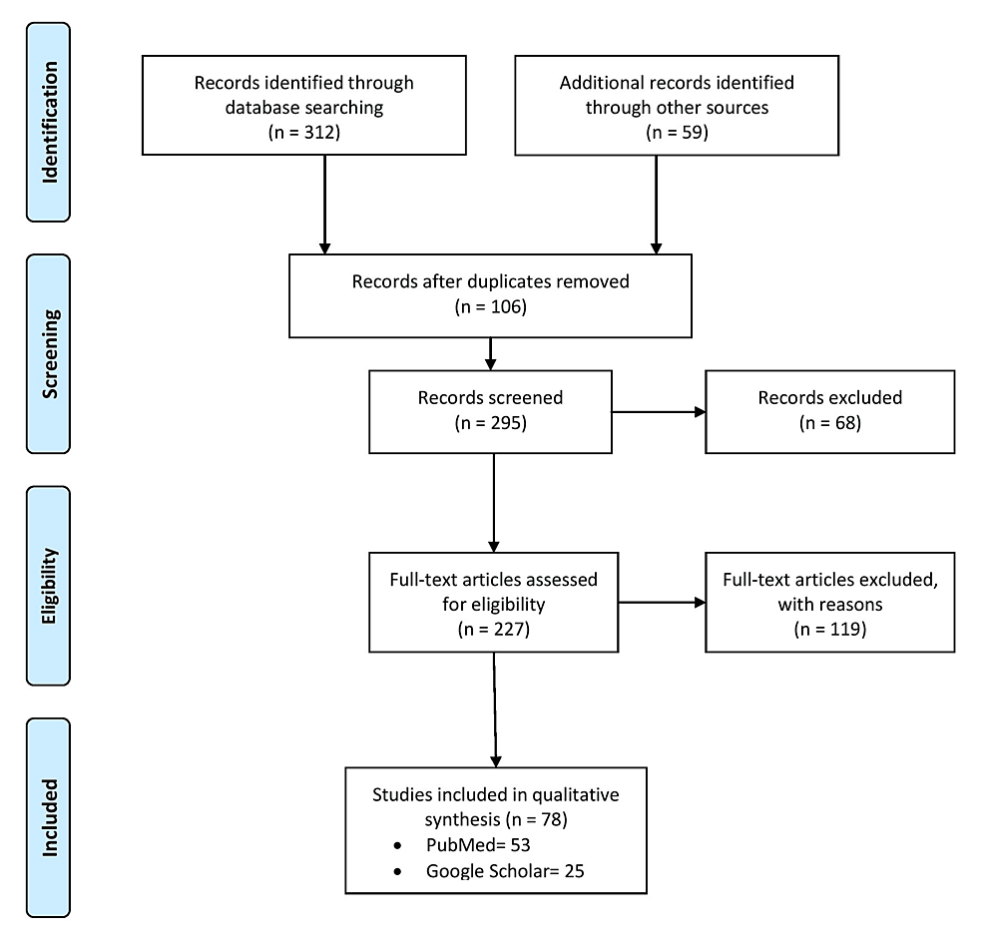
The selection process of articles used in this study Adopted from the Preferred Reporting Items for Systematic Reviews and Meta-Analyses (PRISMA).

The methodology section implemented a comprehensive literature search strategy to gather relevant studies for this review article. Two electronic databases PubMed and Google Scholar were searched using the keywords "anaesthetic considerations," "symptom relief," "multidisciplinary approach," "management strategies," "challenges," "labour," "pregnancy," and "arnold-chiari malformations." The search was limited to English-language articles published in the past decade to ensure the inclusion of recent and pertinent research. Additionally, manual searches of reference lists were conducted to identify additional relevant studies. The inclusion criteria encompassed studies focusing on ACMs in pregnancy and labor, including those that provided insights into the impact of ACMs on maternal health, fetal outcomes, management strategies, anesthetic considerations, and long-term follow-up. Both original research articles and review papers were considered for inclusion. Exclusion criteria involved studies that did not specifically address ACMs in pregnancy and labor, non-English articles, incomplete or unavailable full-text articles, and studies focused on non-pregnant populations or animal models. The selection process involved screening titles, abstracts, and full-text articles to ensure the inclusion of studies meeting the predetermined criteria. Discrepancies in study selection were resolved through consensus among the authors. Figure 1 describes the selection process of articles used in our study.

Overview of Arnold-Chiari malformations

Definition and Classification of ACMs

ACMs are a group of congenital anomalies characterized by structural abnormalities in the posterior fossa of the skull. These malformations involve the displacement of the cerebellar tonsils below the level of the foramen magnum, the opening at the base of the skull. The primary abnormality in ACMs is the herniation of the cerebellar tonsils through the foramen magnum into the spinal canal [6]. Table 1 shows the classification of ACMs and their respective characteristics.

**Table 1:** Classification of ACMs with their respective characteristics ACM, Arnold-Chiari malformations The author recreated the table using the information from [1,7-9]

Type of ACM	Characteristic	Reference
Type I	This is the most prevalent form of ACM and is characterized by the downward displacement of the cerebellar tonsils into the upper cervical spinal canal. Type I ACMs are often associated with a smaller posterior fossa, the space at the back of the skull that accommodates the cerebellum. While some individuals with Type I ACMs may be asymptomatic, others may experience neurological symptoms, such as headaches, neck pain, balance problems, and sensory abnormalities.	[7]
Type II	These are commonly observed in conjunction with myelomeningocele, a spina bifida. In addition to the downward displacement of the cerebellar tonsils, Type II ACMs frequently involve the herniation of other neural structures, including the fourth ventricle and the brainstem. The severity of neurological deficits associated with Type II ACMs can vary, but they often result in more pronounced symptoms and functional impairments than Type I ACMs.	[1]
Type III	These are considered rare and severe. They are characterized by the herniation of cerebellar and brainstem tissue through a defect in the back of the skull. These often present with significant neurological deficits, including profound developmental and intellectual disabilities. Due to the severity of the malformation and associated complications, the prognosis for individuals with Type III ACMs tends to be poor.	[8]
Type IV	They are distinguished by underdevelopment or absence of the cerebellum. Type IV ACM is exceptionally rare and is frequently accompanied by additional abnormalities in the central nervous system. The prognosis for Type IV ACMs is typically guarded and affected individuals often face significant neurological impairments.	[9]

Anatomy and Pathophysiology of ACMs

The anatomy and pathophysiology of ACMs involve the interaction between the posterior fossa structures and the spinal canal. The abnormal position of the cerebellar tonsils can lead to compression and obstruction of the flow of cerebrospinal fluid (CSF) in the region [6]. The displacement of the cerebellar tonsils can result in increased pressure on the brainstem and spinal cord, leading to neurological symptoms. The exact mechanisms underlying the development of ACMs are not yet fully understood, but it is believed to involve both genetic and environmental factors [2].

Prevalence and Risk Factors

The prevalence of ACMs in the general population is estimated to be around 0.1% to 0.5%. However, the true prevalence may be higher due to asymptomatic cases that go undiagnosed. Type I ACMs are the most common form and are frequently identified incidentally during imaging studies for unrelated conditions [10].

The exact risk factors for ACMs are not well-established, but certain factors have been suggested to contribute to their development. These include genetic factors, such as familial clustering, specific genetic mutations, and environmental factors during fetal development [11]. Understanding the prevalence and risk factors associated with ACMs is important for identifying high-risk populations and implementing appropriate screening and management strategies.

Diagnosis of Arnold-Chiari malformations in pregnancy

Challenges and Limitations of Diagnosing ACMs During Pregnancy

Symptom overlap: ACM symptoms, such as headaches, dizziness, and sensory disturbances, can resemble common discomforts experienced during pregnancy. This similarity in symptoms can lead to delayed recognition of ACMs or misattribution of symptoms solely to the physiological changes of pregnancy. Consequently, there is a risk of overlooking the presence of ACMs and delaying appropriate diagnostic evaluation [12].

Limited imaging options: Pregnant women often exhibit concerns regarding the potential risks of invasive or ionizing radiation-based imaging techniques to the developing fetus. Consequently, healthcare providers face limitations in utilizing certain diagnostic modalities commonly employed in non-pregnant individuals. This restriction hampers the ability to obtain detailed anatomical information or definitively confirm the presence of ACMs [13].

Altered anatomy and physiology: Pregnancy itself induces significant changes in the anatomy and physiology of the central nervous system. These changes include increased intracranial pressure and hormonal fluctuations. Such alterations can complicate the interpretation of imaging findings, as they may overlap with ACM-related abnormalities. Distinguishing between changes resulting from pregnancy and those associated with ACMs becomes challenging, requiring careful evaluation and clinical judgment [14].

Imaging Modalities for Diagnosing ACMs in Pregnant Women

When diagnosing ACMs in pregnant women, the choice of imaging modality should prioritize the safety of both the mother and the developing fetus. Non-invasive and radiation-free imaging techniques are preferred to minimize potential risks. In this context, two commonly utilized imaging modalities are magnetic resonance imaging (MRI) and ultrasound [15].

MRI is the gold standard for diagnosing ACMs. It provides detailed anatomical information without ionizing radiation, making it safe for both the mother and the fetus. Precautions should be taken to minimize fetal exposure to the magnetic field by positioning the pregnant woman appropriately during the scan. Additionally, gadolinium-based contrast agents should be avoided during pregnancy due to their potential risks to the developing fetus [16].

Ultrasound is another valuable imaging tool for evaluating ACMs in pregnant women. While it may not provide a definitive diagnosis of ACMs, it is an initial screening tool to assess fetal development and detect structural abnormalities. Ultrasound can help identify associated conditions or anomalies that may be present in conjunction with ACMs. It is a widely available and safe imaging modality that does not involve ionizing radiation, making it suitable for use during pregnancy [17].

The choice of imaging modality may depend on various factors, including the availability of resources, the specific clinical scenario, and the healthcare providers' expertise. In some cases, a combination of MRI and ultrasound may be utilized to obtain a comprehensive evaluation of ACMs and associated conditions in pregnant women. The most appropriate imaging modality should be selected in consultation with the healthcare team and considering the mother's and the developing fetus's specific needs and safety considerations [18].

Screening Recommendations and Guidelines

There are no specific screening recommendations or guidelines for ACMs in pregnancy due to the rarity of the condition and the limitations mentioned earlier. However, considering the potential impact of ACMs on maternal and fetal health, it is important to maintain a high index of suspicion in pregnant women who present with symptoms suggestive of ACMs or have known risk factors [19]. In cases where ACMs are suspected, a multidisciplinary approach involving neurologists, obstetricians, and radiologists is crucial. Close monitoring of symptoms and regular follow-up should be considered throughout pregnancy to ensure appropriate management and timely interventions if necessary [20]. Future research is needed to establish standardized screening protocols and guidelines specifically tailored to diagnosing ACMs during pregnancy. This would help optimize the identification of affected individuals and facilitate appropriate management strategies to mitigate potential risks associated with ACMs in pregnancy.

Impact of Arnold-Chiari malformations on pregnancy

Effects of ACMs on Maternal Health and Well-Being

Neurological symptoms: ACMs can lead to the occurrence or worsening of neurological symptoms, such as frequent headaches, neck pain, dizziness, and sensory disturbances. These symptoms may become more pronounced during pregnancy due to hormonal changes, increased blood volume, and altered intracranial pressure. The interplay of these factors can exacerbate the frequency and severity of headaches and sensory disturbances experienced by pregnant women with ACMs [21,22].

Cardiovascular complications: ACMs can potentially affect cardiovascular function, giving rise to complications such as high blood pressure or cardiac abnormalities. Pregnancy induces physiological changes in the cardiovascular system, including increased blood volume and cardiac output. When combined with ACMs, these changes can further impact cardiovascular health. The altered intracranial pressure associated with ACMs can affect blood pressure regulation, potentially leading to fluctuations and the development of cardiovascular complications [23].

Respiratory difficulties: ACMs may impair respiratory function, particularly during sleep. These malformations can impede the normal flow of CSF, leading to alterations in the pressure within the brain and spinal cord. During pregnancy, changes in lung capacity and respiratory function occur as adaptations to support the growing fetus. These changes can exacerbate respiratory difficulties in pregnant women with ACMs, potentially resulting in sleep disorders and respiratory compromise [24].

Increased risk of trauma: ACMs can contribute to balance and coordination problems, increasing the susceptibility of pregnant women to falls and injuries. The compromised neurological function associated with ACMs can affect motor coordination and proprioception, making pregnant women more prone to accidents and trauma. It is essential to consider the risk of concussion in these individuals and implement preventive measures to ensure their safety [25].

Potential Complications Associated with ACMs During Pregnancy

Pregnancy in women with ACMs carries an increased risk of certain complications due to the interplay between physiological changes of pregnancy and the underlying ACMs. These complications can have significant implications for both the mother and the developing fetus [26]. One potential complication is an increased risk of syringomyelia, frequently associated with ACMs. The formation of fluid-filled cavities within the spinal cord characterizes syringomyelia. During pregnancy, changes in intracranial pressure and spinal fluid dynamics can exacerbate existing syrinx or promote its formation, potentially leading to neurological symptoms and impairments [27].

Neurological symptoms associated with ACMs can be exacerbated during pregnancy. Hormonal fluctuations and increased intracranial pressure can worsen symptoms such as headaches, dizziness, and sensory disturbances. This can increase pain, discomfort, and functional limitations for pregnant women with ACMs [28]. Chiari-related headaches can also become more frequent and intense during pregnancy. These headaches can significantly impact a woman's daily functioning and quality of life, adding to her challenges during this period [29].

ACMs can pose challenges during labor and delivery, increasing the risk of obstetric complications. Prolonged labor, difficult delivery, and the need for interventions such as cesarean section can be more common in women with ACMs. Anesthesia considerations and careful monitoring are crucial to mitigate these risks and ensure the well-being of both the mother and the baby during the birthing process [30].

Impact on Fetal Development and Outcomes

Neural tube defects: Type II ACMs, commonly associated with myelomeningocele, can lead to neural tube defects in the fetus. Neural tube defects are serious congenital anomalies that result in significant neurological impairment and may require specialized care and interventions [31].

Fetal growth restriction: ACMs and associated complications can negatively affect fetal growth, leading to intrauterine growth restriction (IUGR). In these cases, the fetus may fail to reach its expected growth potential, impacting overall health and development. Monitoring fetal growth and well-being is crucial in detecting and managing IUGR [32].

Increased risk of prematurity: Pregnant women with ACMs may face an increased risk of preterm labor and premature birth. This heightened risk can be attributed to factors such as uterine dysfunction, increased susceptibility to complications, and the need for interventions. Premature birth poses potential health challenges for the infant, including respiratory, neurological, and developmental concerns [33].

Fetal distress: ACMs can lead to maternal blood flow and oxygenation alterations, which may impact fetal well-being. This can result in episodes of fetal distress during pregnancy and labor, necessitating close monitoring and timely interventions to ensure the well-being and safety of the fetus [34].

Comprehensive prenatal care, close monitoring of maternal and fetal well-being, and a multidisciplinary approach are essential for managing the potential impact of ACMs on pregnancy and ensuring optimal outcomes for both the mother and the baby. This may involve the collaboration of neurologists, obstetricians, maternal-fetal medicine specialists, and other relevant healthcare professionals [35].

It is important for healthcare providers to be aware of these potential complications and to monitor pregnant women with ACMs throughout their pregnancy closely. Individualized management plans should be developed based on the severity of the malformation, the presence of associated conditions, and the specific needs of the mother and the fetus [36].

Management strategies for pregnant women with Arnold-Chiari malformations

Preconception Counseling and Risk Assessment

Preconception counseling is critical to managing pregnant women with ACMs. It involves a thorough assessment of the woman's medical history, considering the severity of the malformation, associated symptoms, and any previous interventions or surgeries. This comprehensive evaluation aims to provide valuable information for guiding decision-making and optimizing care [37].

The assessment of maternal health is an essential aspect of preconception counseling. It involves evaluating the mother's overall health, including the severity and stability of ACM-related symptoms. The presence of associated conditions, such as syringomyelia (the development of fluid-filled cavities within the spinal cord), is also considered. Understanding the impact of ACMs on daily functioning helps healthcare providers tailor management strategies to address specific needs [37].

Another important consideration during preconception counseling is the evaluation of maternal-fetal risks associated with ACMs. This assessment determines potential risks during pregnancy, labor, and delivery. Factors such as the risk of complications, the impact on fetal development, and the need for specialized care are carefully evaluated. This information assists healthcare providers in developing an individualized management plan that prioritizes the well-being of both the mother and the baby [38]. Given the potential hereditary nature of ACMs, genetic counseling may be recommended as part of preconception counseling. Genetic counseling helps assess the risk of recurrence in future pregnancies and provides appropriate guidance to couples who may be planning to have more children. This aspect of preconception counseling allows for a comprehensive understanding of the genetic factors associated with ACMs and helps individuals make informed decisions regarding family planning.

Multidisciplinary Approach to Care

A multidisciplinary approach involving a team of healthcare professionals is crucial for optimal managing pregnant women with ACMs. This approach recognizes the complexity of ACMs and ensures that expertise from various specialties is utilized to provide comprehensive care. The team may consist of neurologists, obstetricians, maternal-fetal medicine specialists, anesthesiologists, and other relevant specialists, depending on the specific needs of the patient [39].

Collaborative care planning is a key aspect of the multidisciplinary approach. Regular consultations and collaboration among the healthcare professionals involved in the care of the pregnant woman allow for developing a comprehensive management plan. This plan considers maternal and fetal well-being, addressing the unique challenges ACMs pose during pregnancy and labor. By pooling their expertise, the team can create a cohesive and individualized care plan that optimizes outcomes for both the mother and the baby [40].

Close monitoring is another essential component of the multidisciplinary approach. Regular monitoring of maternal symptoms, fetal growth, and well-being throughout pregnancy allows for the early detection of any changes or complications that may require intervention. This proactive approach enables timely adjustments to the management plan, promptly addressing emerging issues. Close monitoring also reassures the patient and her family, promoting a sense of security during this critical period [41].

Effective communication and shared decision-making are integral to the multidisciplinary approach. Open and transparent communication among the healthcare team, the pregnant woman, and her family ensures that everyone is well-informed and actively involved in decision-making. This collaboration allows for informed discussions about treatment options, mode of delivery, and anesthetic considerations, considering the unique circumstances and risks associated with ACMs. By involving the patient and her family in decision-making, healthcare providers can ensure that the chosen interventions align with the patient's values and preferences [42].

Medical Management Options for Symptom Relief

Pain management: Nonsteroidal anti-inflammatory drugs (NSAIDs) and acetaminophen are commonly used for mild to moderate pain relief in pregnant women with ACMs. These medications help alleviate headaches, neck pain, and other discomforts. However, opioids should be used judiciously due to their potential risks and reserved for severe pain unresponsive to other measures [43].

Antiemetic medications: Nausea and vomiting can be exacerbated by ACM-related symptoms during pregnancy. Antiemetic medications may be prescribed to manage these symptoms and provide relief. These medications help reduce the frequency and severity of nausea and vomiting, improving the pregnant woman's comfort [44].

Lifestyle modifications: Simple lifestyle modifications can be beneficial in managing ACM-related symptoms during pregnancy. Adequate rest and proper sleep hygiene can help minimize fatigue and improve overall well-being. Physical therapy exercises, guided by a healthcare professional, may help strengthen muscles, improve posture, and reduce discomfort. Additionally, avoiding triggers exacerbating symptoms, such as certain head positions or activities that increase intracranial pressure, can help manage symptoms effectively [45].

Surgical Interventions and Considerations

Timing of surgery: Generally, surgical intervention for ACMs is deferred until after delivery, if possible. This primarily avoids potential risks associated with anesthesia and surgery during pregnancy. However, surgery may be necessary during pregnancy in situations with severe neurological deterioration or life-threatening complications. The second trimester is often considered the safest period for surgical intervention due to the decreased risk of teratogenic effects on fetal development [46].

Risks and benefits: A thorough discussion between the healthcare team, the pregnant woman, and her family is essential to carefully weigh the risks of surgery against the potential benefits. Risks may include anesthesia-related complications, such as the potential impact on maternal-fetal circulation and the increased risk of miscarriage or preterm labor. It is important to consider the potential benefits of surgery, including symptom relief and improvement in maternal well-being [47].

Fetal monitoring: Close monitoring of fetal well-being before, during, and after surgery is crucial to ensure the safety and well-being of the baby. This may involve fetal ultrasound, non-stress tests, and other appropriate assessments to assess fetal growth, heart rate, and overall well-being. The timing and frequency of these monitoring techniques will be determined by each case's specific needs and the healthcare team's recommendations [48].

Anesthetic Considerations During Labor and Delivery

Anesthetic consultation: Early involvement of an anesthesiologist is crucial to assess the risks associated with ACMs and develop an individualized pain relief plan. The anesthesiologist will evaluate the case, considering the malformation's severity, related complications, and maternal-fetal well-being [49].

Mode of delivery: The delivery method should be determined based on obstetric indications and maternal-fetal well-being. Vaginal delivery is generally preferred as long as there are no specific concerns related to ACMs or associated complications. The decision will consider factors such as the position of the fetus, maternal pelvic anatomy, and potential risks [50].

Pain management during labor: Epidural analgesia or combined spinal-epidural anesthesia may be considered for pain relief during delivery. The anesthesiologist will evaluate the risks and benefits associated with each option, considering the specific case. They will consider factors such as the potential impact on intracranial pressure and the feasibility and safety of administering regional anesthesia techniques [51].

Anesthetic considerations for cesarean section: Regional anesthesia (spinal or epidural) is typically preferred over general anesthesia if a cesarean section is indicated. Regional anesthesia minimizes the risks associated with airway management and reduces the potential increase in intracranial pressure. It allows the mother to remain awake and participate in the birth experience [52].

Postoperative pain management: Adequate pain management is crucial for the mother's comfort and recovery after a cesarean section. Non-opioid analgesics and regional anesthesia techniques can minimize the need for opioids, reducing the potential risks associated with opioid use [53].

Challenges and considerations in labor and delivery

Increased Risk of Complications During Labor and Delivery

Prolonged labor: ACMs can disrupt the normal progression of labor, leading to prolonged labor. Factors such as impaired coordination of pelvic muscles or inadequate uterine contractions can contribute to this challenge. The altered anatomy and function associated with ACMs may impede the efficient progression of labor, requiring additional interventions and prolonged pushing efforts [54].

Difficult delivery: ACMs, particularly type II ACMs associated with myelomeningocele, can increase the risk of difficult delivery. Structural abnormalities, such as a tethered spinal cord or associated neural deficits, can affect the baby's position and descent through the birth canal. These factors complicate the delivery process and necessitate forceps-assisted or vacuum extraction interventions [55].

Increased risk of trauma: Women with ACMs face increased maternal trauma during labor. The balance and coordination issues associated with ACMs can make them more susceptible to falls or injuries, resulting in perineal tears or other trauma. Careful monitoring and appropriate assistance during labor are necessary to minimize the risk of such complications [56].

Increased risk of anesthesia-related complications: Anesthesia considerations during labor and delivery must account for the potential impact of ACMs on airway management and increased intracranial pressure. The presence of ACMs can complicate the administration of anesthesia and pose risks such as difficulty in airway management or worsening of intracranial pressure. Anesthesiologists must exercise caution and expertise in selecting and administering anesthesia techniques to ensure the safety of both the mother and the baby [57].

Vaginal Delivery Versus Cesarean Section: Decision-Making Process

Obstetric indications: The primary consideration for the mode of delivery is the presence of obstetric indications that may necessitate a cesarean section. These indications may include fetal distress, cephalopelvic disproportion (when the baby's head is too large to pass through the mother's pelvis), or other maternal or fetal complications that pose a significant risk during vaginal delivery. In such cases, a cesarean section may be the safest option for both the mother and the baby [58].

Impact of ACMs: The severity of ACMs, associated complications, and the potential risks and benefits of each mode of delivery should be carefully evaluated. The effect of ACMs on the mother's health, such as neurological symptoms or associated conditions like syringomyelia, should be considered. Additionally, the potential risks associated with vaginal delivery, such as increased intracranial pressure or exacerbation of neurological symptoms, need to be weighed against the potential benefits of a vaginal birth [59].

Individual assessment: Each woman's case should be evaluated individually, considering the specific characteristics of her ACMs, associated symptoms, and the risks related to labor and vaginal delivery in her particular situation. Factors such as malformation's severity, symptoms' stability, presence of related conditions, and overall maternal health should be considered. In the absence of obstetric indications, vaginal delivery may be viewed as the primary mode of delivery if it is deemed safe and appropriate for the individual [60].

Monitoring and Management of Pain During Labor

Pain management during labor is a crucial aspect of care for pregnant women with ACMs. Due to the unique challenges ACMs pose, a thoughtful and individualized approach is necessary. Considerations for pain management in this population include non-pharmacological and pharmacological options, as well as the involvement of an anesthesiologist for consultation [61].

Non-pharmacological measures play a significant role in managing labor pain for women with ACMs. Techniques such as relaxation exercises, breathing techniques, position changes, and hydrotherapy can relieve and minimize the need for pharmacological interventions. These methods promote relaxation, reduce tension, and enhance labor comfort [62].

Pharmacological options should be approached with caution, taking into account the potential risks and benefits associated with ACMs and the specific circumstances of each individual. Systemic analgesics, including opioids, may be considered for pain relief. However, the use of opioids should be carefully evaluated due to their potential side effects and impact on the mother and baby. Close monitoring and appropriate dosing are essential [63].

Regional anesthesia techniques, such as epidural analgesia or combined spinal-epidural anesthesia, are effective options for pain management in women with ACMs during labor. Involving an anesthesiologist early in the labor process is crucial to evaluate the feasibility, safety, and potential risks associated with regional anesthesia techniques specific to ACMs. The anesthesiologist will consider factors such as the severity of the malformation, related complications, and potential anesthesia-related concerns when developing an individualized pain management plan [64].

Collaboration among obstetricians, anesthesiologists, and the woman is paramount in determining the most suitable pain management approach during labor. The goal is to provide adequate pain relief while ensuring the mother's and baby's safety and well-being. By utilizing a combination of non-pharmacological measures, pharmacological options when appropriate, and involving an anesthesiologist for consultation, a comprehensive and tailored pain management plan can be developed for pregnant women with ACMs [65].

Case Studies or Real-Life Scenarios Highlighting Challenges and Management Approaches

Including case studies or real-life scenarios in the review article can provide valuable insights into the challenges faced and the management approaches employed in specific cases of ACMs during labor and delivery. These case studies can illustrate the complexity of decision-making, highlight unique challenges encountered, and demonstrate effective management strategies. Case reports or summaries of real-life scenarios can help healthcare professionals better understand the practical implications of managing ACMs during labor and delivery and provide a context for discussion and further research [66,67]. By sharing experiences and outcomes from real-life scenarios, the article can provide a more comprehensive understanding of the challenges and strategies employed in managing ACMs during labor and delivery. It is important to ensure patient privacy and obtain appropriate consent when presenting case studies or real-life scenarios in the article, following ethical guidelines and maintaining confidentiality [68].

Postpartum care and follow-up

Monitoring and Managing Symptoms in the Postpartum Period

Neurological assessment: Regularly monitoring neurological symptoms is essential during the postpartum period. Symptoms such as persistent or worsening headaches, dizziness, or sensory disturbances should be assessed to identify any changes that may require further intervention or management. Ongoing evaluation can help healthcare providers determine the effectiveness of previous treatments and make necessary adjustments to optimize symptom control [69].

Pain management: Continued pain management is crucial for women with ACMs postpartum. This includes the use of appropriate analgesics and non-pharmacological techniques to alleviate discomfort. The choice of analgesics should consider their safety during breastfeeding if applicable. Non-pharmacological approaches such as relaxation techniques, physical therapy, or heat therapy may also relieve pain associated with ACMs [70].

Follow-up imaging: In certain cases, follow-up imaging, such as MRI, may be recommended to assess the malformation status and evaluate any changes or complications that may have occurred during pregnancy or labor. This imaging modality allows healthcare providers to determine the structure of the malformation, the presence of associated complications, or the potential impact of the recent childbirth on the condition. Follow-up imaging helps guide further management decisions and ensures appropriate monitoring of ACMs in the postpartum period [71].

Breastfeeding Considerations

Posture and positioning: During breastfeeding, women with ACMs may experience discomfort or strain on their neck and back. Optimal breastfeeding positions that minimize stress and promote a comfortable latch should be explored. Lactation consultants or healthcare providers can offer guidance on various positions, such as the cradle hold, football hold, or side-lying position, which may be more comfortable for women with ACMs [72].

Support and assistance: Additional support and assistance can benefit women with ACMs regarding breastfeeding. Breastfeeding pillows or cushions can provide extra support for the mother and baby, helping maintain a comfortable position during feeding. Seeking help from a lactation consultant or attending breastfeeding support groups can also provide valuable guidance on proper latch technique and positioning, ensuring effective breastfeeding [73].

Medication considerations: Some women with ACMs may require pharmacological management for ACM-related symptoms, such as pain or neurological symptoms. When considering medications, it is important to assess their safety during breastfeeding. Healthcare providers can provide information on drugs compatible with breastfeeding, weighing the benefits of treatment against any potential risks to the infant. Open communication and consultation with healthcare professionals are crucial in making informed decisions regarding medication use while breastfeeding [74].

Long-Term Follow-up and Management of ACMs in Women Who Desire Future Pregnancies

Before planning a subsequent pregnancy, women with ACMs should undergo preconception counseling. This counseling aims to assess the impact of ACMs on their overall health and well-being and discusses potential risks and management strategies for future pregnancies. It allows one to address concerns, optimize pre-existing management plans, and ensure the woman's readiness for another pregnancy [75].

Risk assessment and monitoring: During subsequent pregnancies, close monitoring of ACM-related symptoms, as well as fetal growth and well-being, is essential. Regular follow-up with healthcare providers, including neurologists and obstetricians, is crucial in ensuring appropriate management and timely interventions if needed. This monitoring identifies any changes in symptoms or ACM-related complications and facilitates prompt intervention or adjustments to the management plan [76].

Genetic counseling: Women with ACMs considering future pregnancies may benefit from genetic counseling. This specialized counseling can help assess the risk of recurrence in subsequent pregnancies and provide guidance on family planning options. It allows for a thorough evaluation of the genetic factors associated with ACMs and assists in making informed decisions about future pregnancies [77].

Multidisciplinary approach: Continued involvement of a multidisciplinary healthcare team is important for the comprehensive management of ACMs during subsequent pregnancies. This team typically includes neurologists, obstetricians, and other relevant specialists. Their collaboration ensures that all aspects of care, such as neurological evaluation, obstetric management, anesthesia considerations, and postpartum follow-up, are addressed in a coordinated manner. This multidisciplinary approach allows for individualized care that optimizes women's overall health and well-being with ACMs and ensures safe and successful subsequent pregnancies if desired [78].

## Conclusions

In conclusion, this review provides a comprehensive overview of the challenges and management strategies pertaining to ACMs in the context of pregnancy and labor. Throughout this review, we have delved into key aspects surrounding ACMs, including their definition, classification, and prevalence. The intricacies of diagnosing ACMs during pregnancy, the available imaging modalities, and the importance of adhering to screening recommendations and guidelines have been thoroughly explored. The impact of ACMs on maternal health and fetal development has been carefully examined, shedding light on the multifaceted nature of this condition during the critical period of pregnancy. The strategies employed for managing ACMs in pregnancy have been discussed comprehensively, emphasizing the vital role of a collaborative and multidisciplinary approach. The concerted efforts of neurologists, obstetricians, anesthesiologists, and various specialists are crucial for the optimal care of pregnant individuals with ACMs. In light of the evidence presented, it is clear that ongoing research in several areas is imperative. Enhanced screening and diagnostic tools, a more profound understanding of long-term outcomes, optimization of anesthesia management, and investigations into genetic and environmental influences are all avenues that warrant further exploration. These research directions hold the potential to advance our knowledge and refine our management strategies regarding ACMs in the unique context of pregnancy and labor. By diligently addressing these research avenues, we can pave the way for improved outcomes for mothers and infants affected by ACMs in pregnancy and labor. This review underscores the significance of evidence-based practices and collaborative efforts in pursuing enhanced care for this distinct patient population.
